# Long-Lasting Nociplastic Pain Modulation by Repeated Administration of Sigma-1 Receptor Antagonist BD1063 in Fibromyalgia-like Mouse Models

**DOI:** 10.3390/ijms231911933

**Published:** 2022-10-08

**Authors:** Beltrán Álvarez-Pérez, Anna Bagó-Mas, Meritxell Deulofeu, José Miguel Vela, Manuel Merlos, Enrique Verdú, Pere Boadas-Vaello

**Affiliations:** 1 Research Group of Clinical Anatomy, Embryology and Neuroscience (NEOMA), Department of Medical Sciences, University of Girona, E-17003 Girona, Catalonia, Spain; 2 WeLab Barcelona, Parc Científic de Barcelona, E-08028 Barcelona, Catalonia, Spain

**Keywords:** fibromyalgia, reserpine, gliosis, sigma-1 receptor, alpha-2-delta-1 subunit, dark/light box test, forced swimming test, Hargreaves plantar test, immunohistochemistry, nociplastic pain

## Abstract

Sigma-1 receptor (σ1R) ligands have been shown to be effective at relieving neuropathic and inflammatory pain, but have not yet been tested in experimental models of fibromyalgia. The objective of this study was to evaluate the effect of a σ1R antagonist (BD1063) compared to pregabalin. ICR-CD1 female mice were subjected to either six repeated injections of reserpine, to cause reserpine-induced myalgia (RIM6), or acidified saline intramuscular injections (ASI). In these two models, we evaluated the effect of BD1063 and pregabalin on thermal hypersensitivity, anxiety-like and depression-like behaviors, and on spinal cord gliosis. BD1063 exerted an antinociceptive effect on both reflexive (thermal hyperalgesia) and nonreflexive (anxiety- and depression-like) pain behaviors, and reduced spinal astroglial and microglial reactivity, following repeated treatment for 2 weeks. Interestingly, the effects of BD1063 were long-term, lasting several weeks after treatment discontinuation in both fibromyalgia-like models. Similar results were obtained with pregabalin, but the effects on pain behaviors lasted for a shorter length of time, and pregabalin did not significantly modulate spinal glial reactivity. The inhibitory and long-lasting effect of pharmacological blockade of σ1Rs on both sensory and affective dimensions of nociplastic-like pain and spinal cord gliosis in two experimental models of fibromyalgia support the application of this therapeutic strategy to treat fibromyalgia.

## 1. Introduction

Pathological pain is a predominant symptom in all fibromyalgia patients, although other symptoms such as fatigue, nonrestorative sleep, mood disturbance, and cognitive impairment are also common, but not universal; such symptoms impact the patient’s quality of life [1,2]. Various pharmacological treatments have been tested in patients with fibromyalgia, including tricyclic antidepressants (e.g., amitriptyline), cannabinoids (e.g., inhaled pharmaceutical-grade cannabis, nabilone), opioids (e.g., hydrocodone, oxycodone, naltrexone, tramadol), gabapentinoids (e.g., gabapentin, pregabalin), serotonin–noradrenaline reuptake inhibitors (e.g., duloxetine, milnacipran), and nonsteroidal anti-inflammatory drugs (e.g., ibuprofen) [1,2,3,4,5]. Only pregabalin, duloxetine, and milnacipran have been approved by the Food and Drug Administration (FDA) in the USA for the treatment of fibromyalgia, while the European Medicines Agency (EMA) has not approved any drugs for this application.

Despite the existence of some treatments, the proportion of treated fibromyalgia patients who achieve worthwhile pain relief is only 10 to 25% higher than with a placebo, and significant pain relief occurs in only 40 to 60% of these patients [5,6]. Hence, new therapeutic options are needed. Discovering new treatments for disease depends on a research continuum usually involving the transition from animal research to clinical studies. One hurdle in drawing meaningful and reliable translational conclusions from animal experiments is the limitation in our ability to model complex diseases with unclear etiology, such as fibromyalgia. Two main models of fibromyalgia-like conditions have been described to mimic altered, long-lasting, reflexive and nonreflexive pain-related behaviors [7]. In an experimental model of reserpine-induced myalgia (RIM3) with three injections of reserpine, the effect of various drugs has been tested in mice, including TRPV1 receptor antagonists [8], serotonin transporters inhibitors [9], bradykinin receptor antagonists [10], ligands of the nociceptin/orphanin FQ receptor [11], dopaminergic receptor agonists [12], toxins [13,14,15], gabapentinoids [14], resolvins [16], and plant-based polyphenol products [17,18,19]. In mice subjected to intramuscular injection of acidified saline solution (ASI), the effect of gabapentinoids [7] and plant-based polyphenol products [20] has also been tested. These studies mainly involved short-term assessment of reflexive responses (i.e., thermal hyperalgesia or mechanical allodynia). However, little is known about the long-term pharmacological effects derived from a more suitable model, such as the RIM6 mouse model, which has been recently described to exhibit long-lasting reflexive and nonreflexive pain abnormalities similar to those in the chronic condition of fibromyalgia [7]. Among possible new pharmacological strategies to alleviate long-term nociplastic pain, we focused on the sigma-1 receptor (σ1R), since there is a growing body of preclinical evidence demonstrating the efficacy of σ1R antagonists in relieving pain in various models of neuropathic and inflammatory pain [21,22,23,24,25,26,27]. To date, this therapeutic strategy has not been assayed in a fibromyalgia-like experimental model. The objective of the present study was to assess and compare the effect of acute and repeated treatment with a σ1R antagonist (BD1063) or pregabalin on reflexive and nonreflexive pain behaviors in two experimental models of fibromyalgia in mice: reserpine-induced myalgia (RIM6) and intramuscular injection of acidified saline solution (ASI).

## 2. Results and Discussion

### 2.1. Sigma-1 Receptor Antagonist BD1063 Exerts Acute Dose-Dependent Antinociceptive Effects in Both RIM6 and ASI Models

Previous studies have shown that σ1R antagonists are able to modulate pathological pain in several preclinical models [21], but their potential modulatory effects on nociplastic pain remain to be investigated. To this end, an acute dose–response (15, 20, 25, 40, or 60 mg/kg) method was designed to determine whether the σ1R antagonist BD1063 exerts antinociceptive effects in two fibromyalgia-like models with long-lasting nociplastic pain: reserpine-induced myalgia RIM6 mice [7,28] and intramuscular acid saline solution injection ASI mice [7,29]. Withdrawal latency to thermal stimulation was assessed immediately before (preadministration) and 30 min after (postadministration) compound treatment. Animals were administered intraperitoneally (i.p.) 28 days after fibromyalgia-like induction in the RIM6 model, or 10 days postinduction in ASI model.

**Figure 1:**
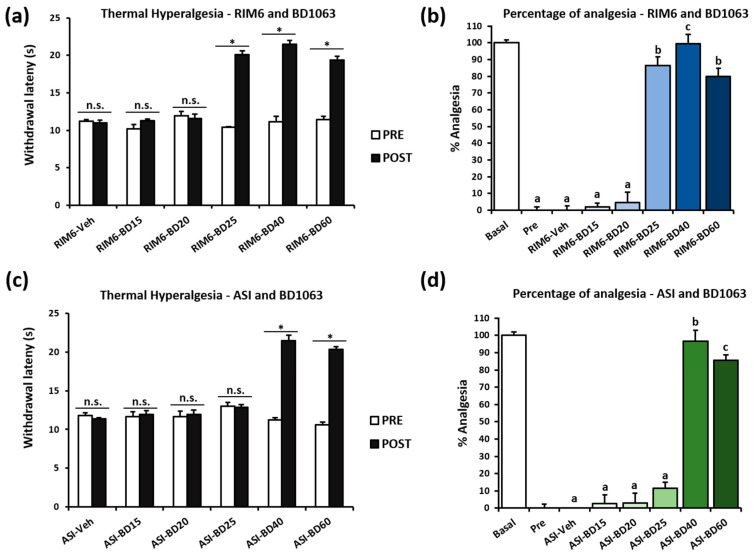
Dose–response effect of BD1063 acute treatment on mice subjected to reserpine-induced myalgia or intramuscular acid saline solution injection. Hind paw withdrawal latency in response to a thermal stimulus before (PRE) and after (POST) BD1063 administration in the (**a**) RIM6 and (**c**) ASI models. Percentage of antinociception 30 min after BD1063 administration in RIM6 (**b**) in ASI models (**d**). Data are expressed as the mean ± SEM (*n* = 5 per group). * *p* < 0.05 PRE vs. POST; n.s. = no significant differences. a–c: Groups not sharing a letter showed significant differences, *p* < 0.05, determined by post hoc tests.

In the RIM6 mice, the dose–response study results showed no significant decrease in hind paw withdrawal latency to thermal stimulation after vehicle administration (*p* = 0.686) or before treatments with BD1063 (*p* = 0.849), i.e., administration of 15 (*p* = 0.225) or 20 mg/kg (*p* = 0.686) of BD1063. In contrast, 25, 40, and 60 mg/kg of BD1063 significantly increased withdrawal latency to thermal stimulation 30 min after administration (all *p*’s < 0.043) (Figure 1a). As shown, when calculating the percentage of antinociception, 25, 40, and 60 mg/kg of BD1063 exerted significant antinociceptive effects when compared with preadministration and vehicle administration (all *p*’s < 0.017), the highest effect being exerted with the 40 mg/kg dose of BD1063 (all *p*’s < 0.05 vs. other effective doses) (Figure 1b). As for the ASI model, no effects were recorded after vehicle administration (*p* = 0.500), before compound administration (*p* = 0.460), or 30 min after administration of 15 (*p* = 0.686), 20 (*p* = 0.686), or 25 mg/kg (*p* = 0.405) of BD1063. Only the 40 and 60 mg/kg doses of BD1063 significantly increased the withdrawal latency to thermal stimulation (all *p*’s < 0.042) (Figure 1c). Similar to the RIM6 model, 40 mg/kg of BD1063 exerted the highest percentage of antihyperalgesic effect in comparison with other doses (all *p*’s < 0.030) (Figure 1d).

These findings suggest that the modulation of the σ1R may be a suitable pharmacological strategy to alleviate nociplastic pain. The results also show that the efficacy of BD1063 is in the range of 25 to 60 mg/kg, the dose of 40 mg/kg being the one exerting maximal antihyperalgesic effects in both animal models. These results are consistent with previous studies using this range of σ1R antagonist administration to reveal antiallodynic and/or antihyperalgesic effects in a variety of rodent models, including spinal cord injury-induced neuropathic pain [22,23], paclitaxel-induced neuropathic pain [24,30], oxaliplatin-induced neuropathic pain [27], partial sciatic nerve ligation-induced neuropathic pain [26], spared nerve injury-induced neuropathic pain [31,32,33], infraorbital nerve constriction injury-induced trigeminal neuralgia [27], carrageenan-induced inflammatory pain [25], and MIA-induced osteoarthritis pain [34].

### 2.2. Repeated Administration of BD1063 and Pregabalin Exerts Long-Term Antinociception in the RIM6 and ASI Mouse Models

According to the above results, a new experiment was designed to determine whether repeated administration of BD1063 at 40 mg/kg, the dose exerting the highest antihyperalgesic effect in both animal models, exerts long-term antinociception (exceeding treatment discontinuation) in fibromyalgia-like models. The effect of BD1063 administered twice a day (i.p.; 6 h between administrations) at 40 mg/kg during 2 weeks after fibromyalgia-like induction was compared with the effect of pregabalin, an FDA-approved drug for fibromyalgia syndrome treatment [35,36], administered once a day at 20 mg/kg, a dose that exerts maximum antinociceptive effects in either RIM6 or ASI models, according to a recent dose–response study in our laboratory [7]. Thermal hyperalgesia was evaluated weekly on a long-term basis, extending to several weeks after the discontinuation of the 2-week treatment. It is worth mentioning that reflexive, evoked pain responses in the RIM6 and ASI models are long-lasting, beyond 6 weeks postinduction in the RIM6 model [7]. Accordingly, antinociceptive effects caused by BD1063 and pregabalin repeat administrations were evaluated on a long-term basis, beyond 6 weeks. As shown below, in addition to describing long-term antinociceptive effects extending several weeks beyond treatment, it was observed that the RIM6 model showed reflexive pain responses until 10 weeks postinduction, whereas for ASI it was only up to 5 weeks postinduction.

**Figure 2:**
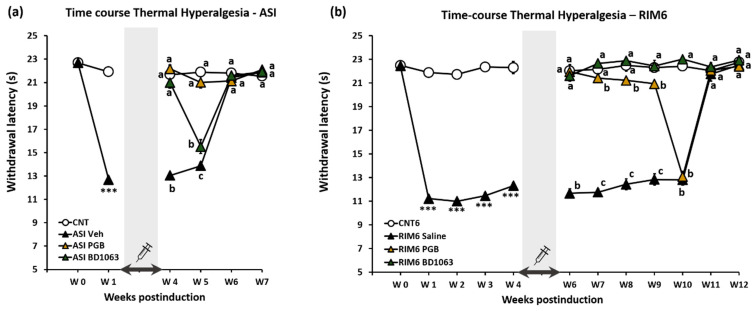
Effect of BD1063, pregabalin (PGB) and saline (vehicle) treatment on the thermal hyperalgesia response in the two experimental models of fibromyalgia. Withdrawal latency to painful thermal stimulus in the intramuscular acid saline solution injection model (**a**) and the reserpine-induced myalgia model (**b**). The shaded bar and the syringe indicate the postinduction period in which pharmacological treatments were administered. *** *p* < 0.001 with respect to the control group. a–c: Groups not sharing a letter are significantly different, *p* < 0.05. Each point and vertical line represent the mean ± SEM.

Following induction by injection of acidic saline solution on the right gastrocnemius muscle (ASI model) on days 0 and 5, thermal hyperalgesia developed from week 1 and lasted up to week 5 from the start of induction (all *p*’s < 0.001; Kruskal–Wallis test). No difference in withdrawal latency to thermal stimulation was observed at weeks 6 (*p* = 0.329) or 7 (*p* = 0.515) in ASI mice as compared to CNT mice (Figure 2a). Accordingly, ASI mice showed significant thermal hyperalgesia before treatment (starting on the fifth day after the last administration of the inducer), during the 2-week treatment period (from day 10 to day 23), and almost 2 weeks after treatment discontinuation (until week 5).

ASI mice that received vehicle administration showed a hind paw withdrawal latency to thermal stimulation significantly lower than control mice (CNT) up to 5 weeks postinduction (all *p*’s < 0.012; Bonferroni’s post hoc test). Subsequently, ASI mice no longer showed significantly decreased withdrawal latency (weeks 6 and 7) in comparison with CNT, indicating that the effect of two intramuscular acid saline solution injections (day 0 and 5) on induction of reflexive pain responses last for 5 weeks (Figure 2a). ASI mice administered either BD1063 or pregabalin for 2 weeks starting on day 10 (5 days after the last administration of the inducer) showed significant increased withdrawal latency to thermal stimulus when compared with ASI-Veh, at 4 (all *p*’s < 0.04 vs. CNT; Bonferroni’s post hoc test) and 5 (all *p*’s < 0.05 vs. CNT; Bonferroni’s post hoc test) weeks postinduction. Hence, repeated administration of BD1063 and pregabalin for 2 weeks exerted long-term antinociception, lasting almost 2 weeks after treatment discontinuation, up to week 5, the limit of the ASI-induced hyperalgesia in the model. When the efficacy was compared, the effect of pregabalin was similar to BD1063 at 4 weeks postinduction (*p* > 0.99; Bonferroni’s post hoc test), but superior to BD1063 at week 5 (*p* = 0.03; Bonferroni’s post hoc test) (Figure 2a).

In the reserpine-induced myalgia RIM6 mouse model, reserpine was administered on days 0, 1, 2, 9, 16, and 23. Thermal hyperalgesia developed from week 1 and lasted up to 10 weeks from the start of reserpine administration. Significantly lower hind paw withdrawal latency to thermal stimulation was shown in RIM6 animals when compared with the corresponding control animals (CNT6) from 1 to 10 weeks postinduction (all *p*’s < 0.001; Kruskal–Wallis test). A lack of significance in withdrawal latency to thermal stimulation was observed at weeks 11 (*p* = 0.494) and 12 (*p* = 0.516), indicating that the effect of the six reserpine administrations on induction of reflexive pain responses last for 10 weeks (Figure 2b). Accordingly, RIM6 animals showed significant thermal hyperalgesia before starting pharmacological treatments (weeks 5–6) up until week 10, 4 weeks after treatment discontinuation.

RIM6 animals administered BD1063 showed significantly increased withdrawal latency to thermal stimulus when compared with RIM6 mice receiving vehicle, from week 6 to 10 postinduction (all *p*’s < 0.017; Bonferroni’s post hoc test). Moreover, no significant differences were recorded during this period when comparing RIM6-BD1063 and CNT6 (Figure 2b). Hence, repeated administration of BD1063 for 2 weeks (weeks 5–6) exerted long-term antinociception, lasting 4 weeks after treatment discontinuation, up to week 10, the limit of hyperalgesia induction in this reserpine-induced myalgia mice model. As for pregabalin administration, RIM6-PGB mice showed significant increased withdrawal latency to thermal stimulus when compared with RIM6-Veh until the week 9 postinduction (all *p*’s < 0.04; Bonferroni’s post hoc test), but no differences were found at week 10 between RIM6-Veh and RIM6-PGB (*p* = 0.635). Moreover, RIM6-BD1063 mice showed significantly higher withdrawal latency to thermal stimuli than RIM6-PGB mice from weeks 7 to 10 postinduction (Figure 2b). These findings indicate that repeated administration of BD1063 exerts superior and longer-term antinociception than pregabalin in RIM6 mice at the assayed doses and administration schedules.

Finally, it is worth mentioning that body weight changes were imperceptible throughout the study, with a maximum of a 1% of loss, and all mice showed similar aspect. Following a protocol for animal welfare supervision based on Morton D.B and Griffiths P.H. guidelines [37], changes in coat and skin, vibrissae of nose, nasal secretions, signs of autotomy in hind paw and/or forepaw, or aggressiveness were not detected after either fibromyalgia-like induction (reserpine or acidified solution administration) or pharmacological treatments (pregabalin or BD1063), indicating that no animal discomfort or abnormal behaviors masking or interfering results were expected in these experiments.

Overall, our findings indicate that repeated treatment with both BD1063 and pregabalin exerts long-term antinociception in fibromyalgia models, lasting several weeks after treatment discontinuation. Some superiority was found for pregabalin over BD1063 in the ASI model (only at week 5), and for BD1063 over pregabalin in the RIM6 model (the effect of BD1063 lasted 1 week more, until week 10, and was somewhat superior at weeks 7–9) at the assayed doses and administration schedules.

Acidic saline intramuscular injection induces the activation of proton sensing ion channels (e.g., ASIC3 and TRPV1) of muscle nociceptors [38,39,40,41,42]. As a consequence of sustained excitation, nociceptive afferent fibers become sensitized, so they release a greater amount of neurotransmitter in the dorsal horn of the spinal cord, which, in turn, overexcites and sensitizes spinal neurons. Similarly, animals treated with repeated administrations of reserpine showed increased sensitivity of nociceptive afferent fibers, mediated, at least in part, by ASIC3 channels. Peripheral hypersensitivity in RIM animals leads to overactivation and sensitization of dorsal horn neurons in the spinal cord, accompanied by decreased inhibitory inputs as well as the activation of spinal microglial cells [43,44,45]. Pregabalin is known to bind to the alpha-2-delta (α_2_δ_1_) subunit of voltage-gated calcium channels, located at the terminals of the nociceptive afferents in the dorsal horn, causing presynaptic inhibition of calcium influx, and thus reduced calcium-induced release of excitatory neurotransmitters, including glutamate [46], thereby reducing the excitability of the spinal nociceptive neurons to relieve pain [47]. Moreover, following peripheral nerve injury, treatment with pregabalin has been shown to enhance norepinephrine release in the spinal cord by the activation of descending inhibitory projections [48]. Accordingly, relief of nociplastic pain by pregabalin potentially involves both inhibition of excitation (i.e., glutamate release from nociceptive afferent fibers) and enhancement of noradrenergic descending inhibition at the dorsal horn of the spinal cord. Interestingly, glutamate N-methyl-D-aspartate (NMDA) receptors associate with α_2_δ_1_ subunits under pathological pain conditions, and this association favors intracellular traffic of NMDA receptors as well as excitatory neurotransmission in the dorsal horn of the spinal cord. Pregabalin treatment reduces the NMDA-α_2_δ_1_ subunit interaction, thus decreasing excitatory transmission in the dorsal horn [49]. In addition to reducing the hyperexcitability of spinal nociceptive neurons, pregabalin could also decrease the reactivity of glial cells, since microglial cells are also sensitive to both glutamate and ATP released by nociceptive afferent fibers [50,51,52,53]. On the other hand, regarding σ1R antagonists, it has been shown that certain antagonist drugs (e.g., NE-100) modulate the release of glutamate by presynaptic nerve terminals [54]. Likewise, the selective σ1R antagonist BD1063 [55] modulates the calcium-dependent release of dopamine and glutamate in striatal nerve terminals [56]. Other selective σ1R antagonists (e.g., E-52862) are also known to stimulate norepinephrine release from descending inhibitory fibers that project towards the dorsal horn of the spinal cord [57]. σ1R is located in the grey matter of the spinal cord. Intense σ1R immunostaining was associated with small- and medium-sized cells located in the superficial layers of the dorsal horn, and less intense immunostaining was associated with other neurons and glia [58]. The σ1R interacts with NMDA receptors and modulates NMDA receptor function [59]. The administration of a σ1R agonist causes neuronal excitability via the activation of the NMDA receptor in the spinal cord [60,61] and phosphorylation of NMDA receptors [62], supporting the observation that σ1R activation promotes excitability and central sensitization of nociceptive neurons in spinal dorsal horn [26]. The application of σ1R ligands also causes the modulation of the physiology of astrocytes [63,64,65,66,67,68,69] and microglial cells [70]. On the contrary, σ1R antagonists inhibit NMDA receptors [59], and the administration of σ1R antagonists inhibits central sensitization [71] and decreases glial reactivity and the production of inflammatory mediators that cause pain [72]. These findings indicate that σ1R modulates neuronal excitability and central sensitization as well as glial cell activation; therefore, the administration of σ1R antagonists (e.g., BD1063) may be used to block a variety of processes involved not only in the induction, but also in the maintenance, of pathological pain. The inhibitory effect on plastic but long-lasting sensitization phenomena once the primary pain-inducing stimuli (i.e., reserpine) have disappeared could thus explain the effect of repeated 2-week BD1063 and pregabalin administration observed 3 (pregabalin) to 4 (BD1063) weeks after treatment discontinuation.

As a mechanistic correlate, given the role of σ1R in neuroimmune and neuroglial interactions during chronic pain [72], and recent data associating spinal cord gliosis with increased thermal hyperalgesia in RIM6 model [7], we investigated the possibility that the antinociceptive effect of BD1063 could be related to such gliosis modulation. These results are explained and discussed in Section 2.3 of the manuscript.

### 2.3. Long-Lasting Antinociceptive Effect of Sigma-1 Receptor Antagonist BD1063 and Pregabalin on Reflexive and Nonreflexive Pain Behaviors Is Accompanied by Spinal Cord Gliosis Modulation in Both Fibromyalgia-like Mice Models

This experiment aimed to investigate spinal cord gliosis modulation as a potential mechanism underlying the effect of BD1063 on nociplastic pain modulation. The experimental design was the same as that used previously, but animals were sacrificed at 10 and 5 weeks postinduction in the RIM6 and ASI model, respectively, in order to obtain spinal cord samples from animals suffering from nociplastic pain at the end of the experiment. Both reflexive and nonreflexive pain behaviors were measured before histological processing. Nonreflexive responses provide complementary information about the overall impact of the persistent sensory abnormality [73,74]. The effect of BD1063 and pregabalin on both anxiety- and depression-like behaviors described in either RIM6 or ASI models [7] were also evaluated by means of the light/dark box and the forced swimming test, respectively.

#### 2.3.1. Long-Term Modulation by BD1063 and Pregabalin of Reflexive and Nonreflexive Pain Responses in Both RIM6 and ASI Models

**Figure 3:**
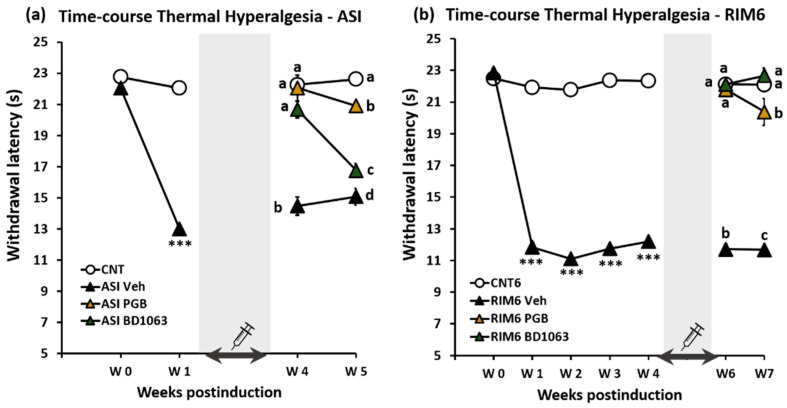
Effect of BD1063, pregabalin (PGB), and saline (vehicle) treatment on the thermal hyperalgesia response in RIM6 and ASI experimental models of fibromyalgia during 7 and 5 weeks postinduction, respectively. Withdrawal latency to painful thermal stimulus (**a**) in the intramuscular acid saline solution injection model (ASI) and (**b**) in the reserpine-induced myalgia model (RIM6). The shaded bar and the syringe indicate the postinduction period in which the different pharmacological treatments have been administered. *** *p* < 0.001 with respect to the control group. a–d: Groups not sharing a letter are significantly different *p* < 0.05. Each point and vertical line represent the mean ± SEM.

Regarding reflexive pain responses, long-term thermal hyperalgesia measured after treatment discontinuation was modulated in both models after either BD1063 or pregabalin repeat administrations for 2 weeks (Figure 3). As expected, in the RIM6 model, both RIM6-BD1063 and RIM6-PGB showed significantly increased withdrawal latency to thermal stimulation at 7 weeks postinduction when compared with RIM6-Veh (all *p*’s < 0.03; Bonferroni’s post hoc test). BD1063 exerted superior antinociception in comparison with pregabalin (*p* < 0.05; Bonferroni’s post hoc test). In the ASI model, at 5 weeks postinduction, ASI-BD1063 and ASI-PGB showed significant antinociception effects when compared with ASI-Veh (all *p*’s < 0.05; Bonferroni’s post hoc test). ASI-PGB showed increased withdrawal latency to thermal stimulation in comparison with ASI-BD1063 (*p* = 0.48; Bonferroni’s post hoc test) (Figure 3).

**Figure 4:**
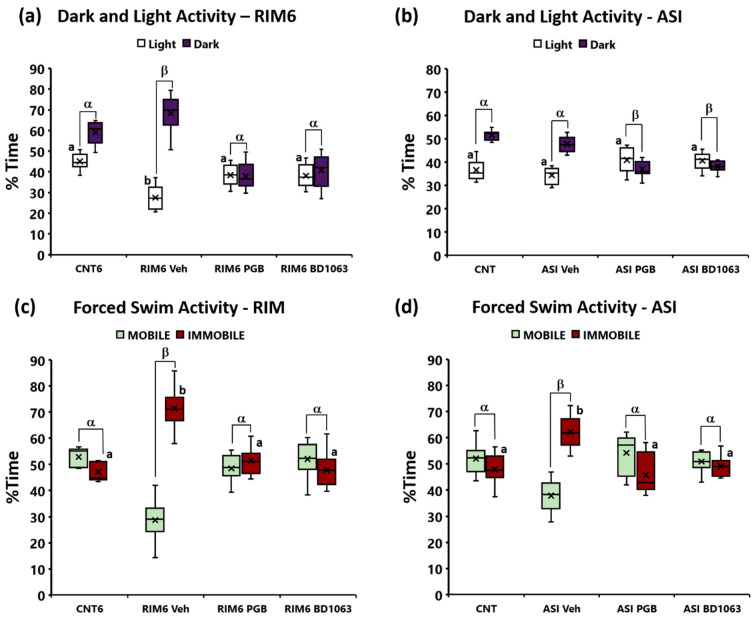
Nonreflexive pain responses in both experimental models of fibromyalgia in animals treated with pregabalin and BD1063. (**a**,**b**) Results of the light/dark box test assessing anxiety-like behavior in both experimental models of fibromyalgia. a,b: Groups not sharing a letter are significantly different in time spent in the light zone, *p* < 0.05. α, β: Groups not sharing a letter are significantly different in time spent in the light zone—time spent in the dark zone, *p* < 0.05. (**c**,**d**) Results of the forced swimming test assessing depression-like behavior in both experimental models of fibromyalgia. a,b: Groups not sharing a letter showed significant differences in %Immobility time, *p* < 0.05. α, β: Groups not sharing a letter showed significant differences in %Immobility–%Mobility time, *p* < 0.05. In all cases, data are expressed as the median ± IQR, and the mean is shown as x.

As for anxiety-like nonreflexive pain responses, significant group differences were found in the light/dark test at 7 weeks postinduction in the RIM6 model (*p* < 0.001; Kruskal–Wallis test) (Figure 4a). RIM6-Veh animals showed a significant decrease in the time spent in the light zone compared with CNT6 animals (*p* < 0.0001; Bonferroni’s post hoc test). In regard to treatments, RIM6-BD1063 and RIM6-PGB increased time the time spent in the light zone compared with RIM6-Veh animals (both *p*’s = 0.004 vs. RIM6-Veh; Bonferroni’s post hoc test), suggesting that both treatments reversed the RIM6 anxiety-like behavior. Moreover, the difference in time spent between dark and light compartments were significantly increased in RIM6-Veh when compared with any of the other groups (all *p*’s < 0.04; Bonferroni’s post hoc test), and both RIM6-BD1063 and RIM6-PGB showed no significant differences when compared with CNT6 (all *p*’s > 0.197; Bonferroni’s post hoc test). In contrast, no differences were observed between groups in the ASI model (*p* > 0.05; Kruskal–Wallis test) (Figure 4b), indicating that anxiety-like behavior did not develop in the ASI model at 5 weeks postinduction, and that treatments did not modulate the time spent in the light zone in ASI mice. Hence, these results indicate that anxiety-like behavior developed at 7 weeks postinduction in the RIM6 model, and was significantly alleviated by both pregabalin and BD1063 treatments, whereas anxiety-like behavior was neither observed at 5 weeks postinduction nor modulated by treatments in the ASI experimental groups.

Depression-like nonreflexive pain response group differences were found both at postinduction week 7 in RIM6 model (*p* < 0.001; Kruskal–Wallis test) and at postinduction week 5 in ASI model (*p* < 0.001; Kruskal–Wallis test). Specifically, the percentage of immobility time of RIM6-Veh was significantly increased when compared with CNT6 (*p* < 0.0001; Bonferroni’s post hoc test) (Figure 4c). Treatments inhibited this depression-like behavior, as both RIM6-BD1063 and RIM6-PGB showed no significant differences in comparison with CNT6 (all *p*’s > 0.425; Bonferroni’s post hoc test). Furthermore, while the difference between immobility and mobility in RIM6-Veh was significantly increased compared with any of the other experimental groups (all *p*’s < 0.004; Bonferroni’s post hoc test), no differences were reported between CNT6 and either RIM6-BD1063 or RIM6-PGB (both *p*’s > 0.425). Similar results were obtained in the ASI model, since the percentage of immobility time of ASI-Veh was significantly increased when compared with CNT (*p* < 0.0001; Bonferroni’s post hoc test) (Figure 4d), and this depression-like behavior was significantly reversed in both ASI-BD1063 and ASI-PGB (all *p*’s > 0.736 vs. CNT; Bonferroni’s post hoc test). Additionally, while the difference between immobility and mobility in ASI-Veh was significantly increased compared with any of the other experimental groups (all *p*’s < 0.005; Bonferroni’s post hoc test), no differences were reported between CNT and either ASI-BD1063 or ASI-PGB (both *p*’s > 0.570). Hence, our results indicate that the depression-like behavior did develop in both RIM6 and ASI fibromyalgia-like mouse models, and that repeated treatments with pregabalin or BD1063 prevented its development.

These results are consistent with previous findings in which animals treated with repeated doses of reserpine showed signs of reflexive pain accompanied by signs of nonreflexive pain (e.g., anxiety or depression) [7,28,75,76,77]. Similar to the present work, pregabalin treatment relieved muscle pain [78] and ameliorated thermal hyperalgesia and/or mechanical allodynia [7,76] in other animal models subjected to reserpine-induced myalgia. Based on our results, depression-like behavior was a predominant sign in both fibromyalgia-like experimental models, whereas anxiety-like behavior developed in the RIM6 model but did not occur in the ASI model. These results are consistent with the conclusions of a recent systematic review in patients with fibromyalgia, indicating that, in addition to pain, depression is a major comorbidity, while anxiety is reported only in a minority of patients [79].

Various supraspinal neuronal nuclei have been involved in a variety of behaviors associated with pain, especially depression- and anxiety-like behaviors. It has been reported in nerve-injured animals that, in the short term, the injury induces pain and ipsilateral locus coeruleus (LC) activation, while in the long term there is bilateral LC activation [80]. The inhibition of the LC ipsilateral to the nerve injury leads to a reduction in pain intensity, but the inhibition of the contralateral LC does not modify the sensation of pain. When bilateral LC inhibition occurs, a decrease in the depressive response is observed in long-term nerve injury, suggesting that unilateral LC activation reduces pain, whereas bilateral LC activation leads to the development of a depressive response associated with pain. Likewise, in sham animals, the chemogenic activation of the LC-ipsilateral or LC-contralateral triggers the appearance of depressive responses in sham animals. In the experimental model of chronic trigeminal neuralgia, the parabranchial nucleus (PBN)–ventral tegmental area (VTA) pathway is involved in the development and production of depressive responses in chronic pain states. Inhibition of VTA dopaminergic neuron activity alleviates depressive-like behavior, and the blockade of glutamate release by parabranchial nucleus afferents on VTA neurons also alleviates depressive-like behaviors [81]. On the other hand, stimulation of GABAergic projections from the central nucleus of the amygdala to glutamatergic neurons of the PBN of the thalamus causes an increase in depressive behaviors in mice subjected to chronic restraint stress, while its inhibition alleviates these depressive responses [82]. Activation of the lateral habenula in chronic constriction of the sciatic nerve triggers mechanical hyperalgesia and depression, suggesting an involvement of the habenula in neuropathic pain and accompanying depressive-like behavior [83]. In this same nerve injury experimental model of neuropathic pain, injury to the lateral habenula caused relief of mechanical allodynia and depression-like responses, accompanied by the activation of neurons in the dorsal raphe nucleus, suggesting that the lateral habenula and dorsal raphe, two anatomically and functionally related areas, play important roles in the regulation of pain and depression-like responses [84]. Inhibition of the projections from the dorsal raphe to the central nucleus of the amygdala produced depression-like behavior in a male mouse model of chronic pain, whereas activation of this pathway using pharmacological or optogenetic approaches reduced depression-like behavior [85]. Finally, in experimental models of neuropathic and/or inflammatory pain, it has been shown that stimulation of the projections of the basolateral nucleus of the amygdala to the central nucleus of the amygdala inhibits anxiety and pain [86]; and chemogenic inhibition of medial septum neurons, as well as medial septum projections towards the rostral anterior cingulate cortex, inhibits pain-associated anxiety [87]. Spinal nociceptive neurons project upward to LC neurons [88]. The PBN, particularly the lateral PBN (lPBN), has an important role in receiving, processing, and relaying somatic and visceral nociceptive signals [89,90]. It is the primary supraspinal target of nociceptive transmission from spinal superficial and deep dorsal horn neurons [91,92,93,94,95,96]. In rodents, 95% of lamina I projection neurons in the spinal cord project towards the PBN [97]. Indirect connections of spinal nociceptive neurons with the neurons of the lateral habenula, through the raphe nuclei and the LC, have also been described, as well as synaptic connections between neurons of the raphe nuclei and neurons of the lateral habenula [98,99] and the LC and the lateral habenula [98]. Via the lateral habenula, spinal nociceptive signals can reach the thalamic PBN [100], although the nucleus raphe magnus also projects spinal nociceptive information to the thalamic PBN [101].

Taken together, it is clear that a variety of supraspinal nuclei provide a neuroanatomical substrate for common neurobiological mechanisms of pain and depression, which may explain the coexistence of pathological pain and depression. This coexistence between pain and depression has also been observed in the present study in fibromyalgia-like animal models.

#### 2.3.2. BD1063 Treatment Significantly Reduced Spinal Cord Gliosis in RIM6 and ASI Animals

**Figure 5:**
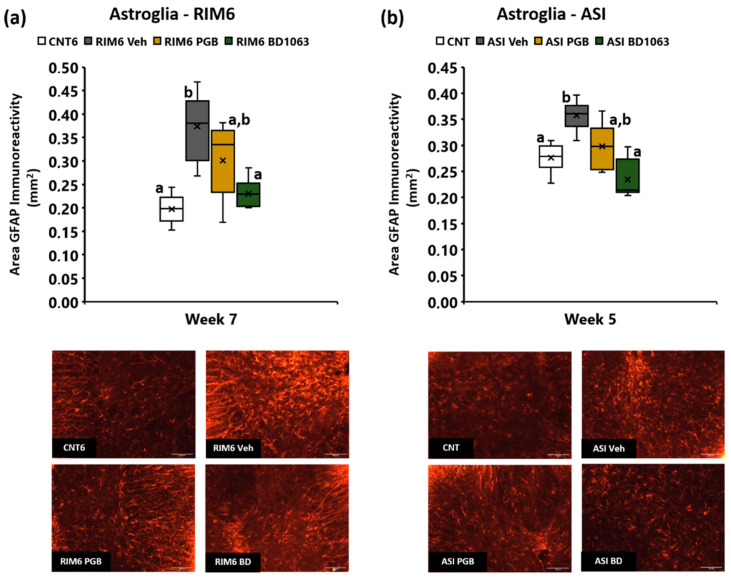
Spinal cord astrogliosis in mice subjected to (**a**) reserpine-induced myalgia and (**b**) intramuscular acid saline solution injection, and treated with saline solution, BD1063, or pregabalin. Representative histological images of the spinal cord immunostained for GFAP of each group (scale bar 100 μm) are shown in the bottom of the figure. Data are expressed as the median ± IQR, and the mean is also shown as x. a,b: Groups not sharing a letter showed significant differences respect to control group, *p* < 0.05.

Spinal cord gliosis was evaluated in order to gain mechanistic insights. Regarding astrogliosis, significant differences in GFAP immunoreactivity were found at postinduction weeks 7 (*p* <0.001; Kruskal–Wallis analysis) in RIM6 animals (Figure 5a), and BD1063 reversed astrogliosis was observed in RIM6-Veh animals (*p* = 0.006; Bonferroni’s post hoc test). As for pregabalin, RIM6-PGB did not significantly differentiate from either RIM6-Veh (*p* = 0.276) or RIM6-BD1063 (*p* = 0.270), indicating that its effect on astrogliosis did not reach statistical significance. Similar results were obtained in the ASI model. Astrogliosis developed in ASI-Veh 5 weeks postinduction, and astrogliosis was reversed in ASI-BD1063 as compared with ASI-Veh (*p* = 0.001; Bonferroni’s post hoc test), but ASI-PGB did not significantly differentiate from either ASI-Veh (*p* = 0.365) or ASI-BD1063 (*p* = 0.628) (Figure 5b).

**Figure 6:**
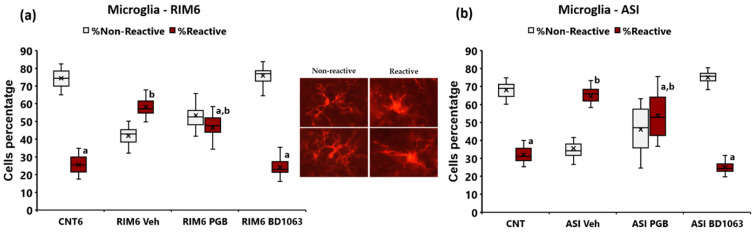
Spinal cord microgliosis in mice subjected to (**a**) reserpine-induced myalgia and (**b**) intramuscular acid saline solution injection, and treated with saline solution, BD1063, or pregabalin. In the middle, representative nonreactive and reactive microglial cells. Note that reactive cells have an amoeboid form, a larger nucleus, and shorter branching processes than nonreactive cells. Images were captured at regions subjected to astrogliosis analysis. Data are expressed as the median ± IQR, and the mean is shown as x. a,b: Groups not sharing a letter showed significant differences with respect to control group, *p* < 0.05.

Regarding spinal cord microgliosis, significant differences in the percentage of reactive microglia were found in RIM6 animals (*p* < 0.001; Kruskal–Wallis analysis) (Figure 6a) and ASI mice (*p* < 0.001; Kruskal–Wallis analysis) (Figure 6b). Specifically, RIM6-Veh showed significant increase in the percentage of reactive microglia in comparison to CNT6 (*p* < 0.0001; Bonferroni’s post hoc test), and this microgliosis was reversed after BD1063 administration (*p* = 0.887 vs. CNT; Bonferroni’s post hoc test). In contrast, RIM6-PGB did not significantly differentiate from either RIM6-Veh (*p* = 0.316) or CNT6 (*p* = 0.827), indicating that pregabalin did not significantly modulate microgliosis (Figure 6a). Similarly, while ASI-Veh showed a significant increase in the percentage of reactive microglia in comparison to CNT (*p* = 0.008; Bonferroni’s post hoc test), this microgliosis was reversed after BD1063 administration (*p* = 0.920 vs. CNT; Bonferroni’s post hoc test). Regarding pregabalin, ASI-PGB did not significantly differentiate from either ASI-Veh (*p* = 0.394) or CNT6 (*p* = 0.121) (Figure 6a).

Our findings suggest that long-term antinociceptive effects of repeated administration of BD1063 may be associated with both astro- and microgliosis downregulation in the spinal cord in the RIM6 and ASI models. This is the first study supporting a role of σ1R in the modulation of spinal gliosis in fibromyalgia-like experimental models. The results obtained with pregabalin do not support a modulatory role of this compound in modulating glial reactivity in the spinal cord in fibromyalgia-like models. The difference between BD1063 and pregabalin could be related to the expression of their respective molecular targets, as α_2_δ_1_ subunits have not been described in astrocytes and microglial cells; however, σ1R is expressed in glial cells, both in astrocytes [63,65,69,102,103,104,105] and microglial cells [70,106,107,108,109,110,111]. In this way, as reported in other models of pathological pain, such as chronic constriction injury, there is an upregulation of σ1R expression in spinal cord astrocytes, accompanied by overexpression of GFAP. Blockade of σ1R with the σ1R antagonist BD1047 relieved pain-related behaviors and significantly reduced astrocyte reactivity, accompanied by a reduction in phospho-p38-MAPK expression in astrocytes [63]. The σ1R also modulates the release of D-serine by reactive astrocytes, which contributes to mechanical allodynia after nerve injury, and D-serine release was drastically reduced when BD1047 was administered, which also reversed mechanical allodynia [64]. In vitro stimulation of σ1R with a selective agonist (e.g., SKF-10047) caused proliferation, migration, and overexpression of GFAP in astrocytes. All these changes were reversed with a σ1R antagonist (e.g., BD-1047) [104]. Likewise, the administration of a σ1R agonist (e.g., PRE-084) induced an influx of calcium ions in astrocyte cultures, which was reversed by applying a σ1R antagonist (e.g., BD1047). In animals subjected to chronic constriction of the sciatic nerve, intrathecal administration of PRE-084 induced thermal hyperalgesia and mechanical allodynia, along with overexpression of σ1R and GFAP in the dorsal horn of the spinal cord. All these functional and molecular changes were reversed with the intrathecal administration of BD1047 [69]. Taken together, these findings suggest that σ1R ligands modulate astrocyte physiology; agonists activate astrocytes while antagonists inhibit this activation. In experimental models of neuropathic pain, the administration of σ1R antagonists reduced pain responses, and this was accompanied by a reduction in spinal astrogliosis, similar to the observations in the present study using experimental models of fibromyalgia.

As for microgliosis modulation, the LPS-stimulated murine BV2 microglia cells secrete various proinflammatory factors such as TNF-alpha, IL-1 beta, and oxygen free radicals, and the addition of σ1R antagonists (e.g., BD1047, BD1063) to cultures of LPS-reactive microglia cells inhibited the secretion of these proinflammatory factors [109]. Moreover, following intraplantar injection of inflammatory agents such as complete Freund’s adjuvant (CFA) and zymosan, the administration of BD1047 inhibited thermal hyperalgesia, mechanical allodynia, and spinal microglial cell reactivity [112,113]. In the experimental model of bone cancer pain, administration of BD1047 alleviated mechanical allodynia and reduced microglial reactivity and TNF-alpha secretion [70]. Accordingly, σ1R ligands are also suggested to modulate microglia cell physiology, which correlates with attenuation of pain and inhibition of secretion of proinflammatory factors.

The σ1R is a small transmembrane protein (25 KDa) localized predominantly at the mitochondrion-associated membranes (MAM) in the endoplasmic reticulum (ER). In the region of the endoplasmic reticulum σ1R regulates the flow of calcium via inositol 1,4,5-trisphosphate (IP3) receptors, acting as a ligand-operated molecular chaperone. Via molecular chaperone activity, the σ1R regulates protein folding/degradation, oxidative stress, and cell survival. Under cellular stress, there is a σ1R translocation from the ER-MAM to the plasma membrane, where it joins a variety of transmembrane proteins such as neurotransmitter receptors and ion channels, exerting modulatory effects on these transmembrane proteins. These modulatory effects will be excitatory or inhibitory since σ1R can bind various substances that act as agonists (e.g., PRE084) or antagonists (e.g., BD1047, BD1063) [114,115,116,117].

In addition to the mechanisms described above for the effect of pregabalin and BD1063 treatment on nociplastic pain, it is also known that σ1Rs are located in the nociceptive afferent fibers that project into the dorsal horn of the spinal cord. In these afferent nerve fibers, σ1R modulates TRPV1 receptors activated by capsaicin because peripheral capsaicin injection is unable to induce mechanical hyperalgesia in KO animals for σ1R, and this effect is also mimicked in wildtype animals treated with BD1063 [118]. Experimental evidence suggests that σ1R antagonists reduce the association of the σ1R with the C-terminal part of the TRPV1 receptor, while agonists favor it [119]. Furthermore, σ1R also interacts with ASIC channels [120] and modulate the proton-gated ion channels [121] located in afferent nociceptive fibers. In this context, treatment with σ1R antagonists has been shown to reduce mechanical allodynia synergistically with ASIC channel blockers [122]. Together, the σ1Rs modulate transmembrane protein complexes located in the nociceptive afferent fibers, and thus the hyperexcitability of these primary afferent neurons. TRPV1 and ASIC receptors are involved in the hyperexcitability of nociceptive afferent fibers in both experimental models of fibromyalgia, as previously indicated.

In addition to interacting and modulating NMDA receptors as described previously, it is known that σ1Rs also modulate adrenergic receptors. Administration of clonidine, an α-2-adrenergic receptor agonist, attenuates hyperalgesia. These analgesic effects are enhanced when there is a coadministration of clonidine and a σ1R antagonist [123]. These α-2-adrenergic receptors are located on spinal nociceptive neurons postsynaptically to nociceptive afferent fibers that penetrate the dorsal horn [124], and are involved in the inhibition exerted by norepinephrine, released by descending projections, on spinal nociceptive neurons [125,126]. In addition, σ1R antagonists (e.g., E-52862) promote norepinephrine release in the dorsal horn of the spinal cord [57]. These pieces of preclinical evidence suggest that σ1R antagonists, via σ1R binding, enhance the inhibitory effects exerted by norepinephrine and α-2-adrenergic receptor agonists on spinal nociceptive neurons. Therefore, σ1R antagonists potentiate the inhibition exerted by the descending pathway on spinal neurons. This effect of σ1R antagonists on potentiating hyperpolarization caused by norepinephrine binding to α-2-adrenergic receptors on spinal neurons is important in both experimental models of fibromyalgia, since it reduces the hyperexcitability of spinal neurons to peripheral inputs of hyperexcited afferent fibers in both fibromyalgia models (ASI and RIM6), as well as in the reserpine-induced myalgia model (RIM6), where reserpine causes a reduction in levels of norepinephrine released by descending projections in the dorsal horn [28,43]. Therefore, σ1R antagonist treatment makes the low levels of norepinephrine released at descending synapses more effective at inhibiting spinal neurons.

NMDA receptor stimulation in primary cultured microglia induces their proliferation, morphological activation, and release of proinflammatory mediators via PARP-1/TRMP2 signaling [127]. It is well known that in situations of cellular stress, when intracellular calcium increases, σ1R is activated and translocated to plasma membrane, where is physically interacts with several membrane proteins, including the receptors listed above as well as NMDA receptors [72]. Moreover, σ1R agonists increase and σ1R antagonists decrease NMDA receptor calcium flow through the channel [71], and the calcium influx activates several signaling pathways implicated in the synthesis and release of proinflammatory mediators, such as PARP-1/TRMP2 [127] and p38-MAPK [128]. On the other hand, TRPV1 receptors present on microglia cells, functioning as a calcium influx-regulating channel, play an important role in NLRP3 inflammasome activation of these glial cells [129]. As described above, σ1R modulates the TRPV1 channel [118,119,130], and consequently, it may also regulate the inflammasome of microglial cells. Experimental evidence also shows that σ1R modulates the activity of purinergic receptors (e.g., P2X) [122]. Microglia cells also express P2X4 and P2X7 receptors, which are involved in the activation of signaling pathways (e.g., p38-MAPK), that generate proinflammatory mediators that cause pain [52,131,132]. These findings suggest that σ1Rs may also regulate purinergic receptors on microglial cells, and thus the synthesis and release of proinflammatory mediators of pain.

In contrast to σ1R ligands that can bind to these receptors located on the ER-MAM as well as on the plasma membrane, pregabalin is a ligand for the σ_2_δ_1_ subunit of voltage-gated calcium ion channels [133], preferentially located in the plasma membrane. Pregabalin inhibits glutamate release from the synaptic endings of nociceptive afferent fibers in the dorsal horn of the spinal cord [47], and also inhibits trafficking of the calcium channel α_2_δ_1_ subunit to presynaptic terminals [134]. Ligands of σ_2_δ_1_ subunit also modulate the release of neurotransmitters from the descending noradrenergic system. Specifically, pregabalin activates the release of norepinephrine by this descending system on the nociceptive neurons of the dorsal horn [48]. On the other hand, in microglia cells, pregabalin mitigates inflammatory responses by directly inhibiting cytoplasmic translocation of high-mobility group box 1 (HMGB1), and consequently reduces the synthesis and release of proinflammatory mediators [135]. In addition, microglia cells express L-type voltage-gated calcium ion channels [136], and these calcium channels also express the σ_2_δ_1_ subunit that modulates its functionality [137]. The influx of calcium ions into microglia cells favors the synthesis and secretion of proinflammatory cytokines [138]. These findings suggest that pregabalin, by blocking the entry of calcium ions through L-type channels, may prevent the generation of inflammatory cytokines in microglia cells.

Similar to our results from fibromyalgia-like models involving nociplastic pain, the administration of σ1R ligands causes the relief of thermal hyperalgesia and mechanical allodynia, and this is accompanied by a reduction in glial reactivity in different models of neuropathic pain and inflammatory pain. Glial activation is accompanied by the secretion of proinflammatory factors (e.g., cytokines, chemokines, prostaglandins) that contribute to increased pain neurotransmission in the dorsal horn of the spinal cord, and to increased sensitization of spinal nociceptive neurons, thus enhancing the hyperexcitation of spinal nociceptive neurons and, therefore, pain responses [139,140]. Treatment with σ1R antagonists may constitute an effective pharmacological therapy to reduce glial reactivity and levels of pain-promoting chemicals, thereby alleviating reflexive and nonreflexive pain responses.

## 3. Materials and Methods

### 3.1. Animals

Adult female CD1 mice (22–26 g) aged 8 weeks were obtained from Janvier Laboratories (France). They were housed with access to both food and water ad libitum in a colony room at 19–22 °C and 40–60% humidity under a 12:12 h light/dark cycle in groups of five in appropriate plexiglass cages with wood-shaving bedding. Cages were changed twice weekly. All mice were allowed to acclimatize for at least 1 h to the facility rooms before functional, behavioral, and administration procedures, which were all conducted during the light cycle. Sentinel mice were routinely tested for pathogens, and facilities remained pathogen-free during the whole experimental period. The number of mice used in this study was maintained at a minimum, working with experimental groups consisting of 6 to 12 mice. The animal sample size was calculated using GRANMO (Version 7.12 April 2012) and based on the ethical limits proposed by the Animal Ethics Committee.

All experimental procedures and animal husbandry were conducted following the ARRIVE 2.0 guidelines and according to the ethical principles of the IASP for the evaluation of pain in conscious animals [141], as well as the European Parliament and the Council Directive of 22 September 2010 (2010/63/EU). The procedures were also approved by the Animal Ethics Committee from the University of Barcelona (CEEA: 35/16; DAMM: 8887). All efforts were made to minimize animal suffering and to keep the number of animals to a minimum to demonstrate consistent effects for the procedures and treatments.

### 3.2. Induction of Fibromyalgia-like Condition: RIM6 and ASI Models

Two mouse models of fibromyalgia-like conditions were used in this study: reserpine-induced myalgia RIM6 mice [7,28] and intramuscular acid saline solution injection ASI mice [7,29]. Briefly, for the RIM6 model, reserpine (Sigma-Aldrich; St. Louis, MO, USA) dissolved in acetic acid and diluted to a final concentration of 0.5% acetic acid with saline solution was administered subcutaneously (0.25 mg/kg) on days 0, 1, 2, 9, 16, and 23. ASI mice were injected with acid saline solution (pH = 4, 10 µL) into the right gastrocnemius muscle, using a Hamilton syringe, on days 0 and 5. Following the same schedule of administrations, the corresponding control animals received either subcutaneous administration of reserpine dilution vehicle (CNT6 group) or intramuscular saline solution injection (CNT group).

### 3.3. Pharmacological Assessment

The pharmacological assessment was divided into three independent experiments. The first consisted of conducting a dose–response study to evaluate the acute antinociceptive effects of BD1063 in both RIM6 and ASI models. To this end, 5 days after the last administration of the inducer (reserpine or acidified solution), a set of mice (*n* = 5 per group) were treated with BD1063 (i.p.) at doses of 15, 20, 25, 40, or 60 mg/kg, and control CNT6 and CNT mice received the vehicle solution. Thirty minutes after administration, thermal hyperalgesia was evaluated as described below. The second experiment was designed to determine long-term antinociceptive effects of BD1063 in both models and compare its effects with pregabalin administration, an FDA-approved drug for FMS treatment [35,36] that exerts short-term thermal hyperalgesia modulation when administered acutely in the RIM and ASI models [7]. During 2 weeks starting from the fifth day after the last administration of the inducer, another set of animals were administered twice a day (i.p.; 6 h between administrations) using the BD1063 dose that exerted better antinociceptive effects in the dose–response study (40 mg/kg), or once a day using the most suitable dose of pregabalin (20 mg/kg) according to recent results [7]. The thermal hyperalgesia of animals of this new set (*n* = 6–10 per group) was evaluated once a week until nociceptive response of either untreated RIM6 or ASI animals was no longer present, indicating the limits of the fibromyalgia-like induction models. Finally, in order to gain mechanistic insights, both astrogliosis and microgliosis in the spinal cord were studied in a third set of animals (*n*= 11–15 per group); RIM6 and ASI mice were administered following the scheduled induction and treatment processes explained above, and after evaluating thermal hyperalgesia once a week, the mice were sacrificed within the window in which both models still showed nociplastic pain responses (7 and 5 weeks postinduction in RIM6 and ASI, respectively), and BD1063 and pregabalin pharmacological effects were still present. That is, spinal cord samples were collected 2 weeks after repeated administration in both models. Moreover, during this experimental period, the anxiety- and depression-like behaviors were evaluated by means of dark/light and forced swimming tests, as nonreflexive pain responses.

### 3.4. Functional Evaluation

#### 3.4.1. Reflexive and Nonreflexive Pain Responses Assessments

Thermal hyperalgesia, as reflexive pain response, was determined according to procedures explained elsewhere [7,22,23,142,143], using the plantar thermal algesimetry or plantar test (#37370; Ugo Basile, Comerio, Italy). Mice were placed inside a methacrylate box on top of a platform with a glass floor. Before starting the test, the animals were allowed to acclimatize for 45 min. A radiant heat source (100 W) was then positioned under the plantar surface of the animal’s hind paw and activated. The time of paw withdrawal to this thermal stimulus was recorded. Each hind paw was evaluated three times separately, leaving a rest time of 5 min between evaluations. A maximum limit of 25 s was imposed to prevent tissue damage in the absence of a withdrawal response. The final value of each animal was the average of the values of the withdrawal time of both paws in the three times.

The anxiety-like behavior response was evaluated by means of the dark/light box test, and the depression-like behavior was evaluated by the forced swimming test. The dark/light box test consisted of an illuminated 27 × 27 × 26 cm white compartment with an open top connected via an opening entrance (5 × 5 cm) to a 27 × 27 × 26 cm black box compartment (covered with a lid). Mice were placed in the dark compartment and allowed to freely explore the apparatus for 10 min, and the time spent in the light compartment and the latency to enter the light compartment were recorded. The room light was 45–50 lux and the whole session was recorded for analysis using Panlab Smart video tracking software (SMART V3.0.06; Harvard apparatus, Barcelona, Spain). After each 10 min trial, both compartments were cleaned with 5% ethanol/water [7,144]. The forced swimming test, which it is known to be a useful procedure for depression-like behavior evaluation [7,145,146], was performed in open cylinders (40 cm height × 15 cm diameter) containing 30 cm of water at 25 ± 1 °C, in which mice were individually forced to swim. Their mobility and immobility time during the 6 min test was recorded with a video camera (Sony HDRCX190). The immobility time was determined whenever no additional activity other than the necessary movements to keep the mouse’s head above the water was observed.

#### 3.4.2. Histological Evaluation

At the end of the functional and behavioral evaluation, the animals were deeply anesthetized with sodium pentobarbital (90 mg/kg; i.p.). Then, the spinal cord was removed and the spinal segment distal to T10 was placed in an Eppendorf tube with Zamboni’s fixative solution for 15 days [147]. Subsequently, the fixative solution was changed for another cryoprotective solution based on 30% sucrose in phosphate buffered saline (PBS; 0.1 M; pH = 7.4). The spinal cord segment remained in the cryoprotective solution until it was well impregnated, which took at least another 15 days. The spinal cord segment was then cut into 60 µm thick sections using a cryostat (CM1520, Leica, Barcelona, Spain). To achieve this, each spinal cord segment was embedded in a cryoprotective solution of tissue freezing medium (Ref: 0201-08-926; Leica, Barcelona, Spain), which was frozen until a block with the spinal cord segment was formed. The cross-sections were collected in 6-well porcelain plates. It should be noted that all the blocks began to be cut at level T10 and in a caudal direction. Then, under constant agitation, two washes were performed with phosphate buffered saline (PBS; 0.1 M, pH = 7.4) for 10 min each, followed by two more 10 min washes with 0.3% Triton-X-100 in PBS (PBS-Triton), and finally another 45 min wash with 1% fetal bovine serum in PBS-Triton (PBS-Triton-FCS). After bathing, the histological sections were incubated with primary antibodies rabbit anti-glial fibrillary acidic protein (GFAP; 1:200; ab7260, ABCAM, Cambridge, UK) to visualize the astrocytes, and rabbit anti-adaptor molecule 1 ionized calcium-binding agent (Iba1; 1:200; 019-19-741; WAKO, Richmond, VA, USA) to visualize microglia cells. PBS-Triton-FCS was used as a diluent for the primary antibodies. The sections were kept incubating with these primary antibodies between 48–72 h, at 4 °C, in a humidified chamber and under constant agitation. Subsequently, and after several washes with PBS-Triton, the histological sections were incubated at 4 °C for 24 h with donkey anti-rabbit secondary antibodies conjugated with cyanine 3.18 (1:200, Cy3, Jackson Immnoresearch, West Grove, PA, USA), in a humidified chamber and under constant agitation. Finally, after several washes with PBS-Triton and PBS, the sections were mounted on pregelatinized slides, dehydrated in ethanol solutions (70°, 96°, absolute ethanol) and the coverslips were mounted with DPX (Ref: 1.01979.0500, Merck, Germany). As a specificity control, some spinal cord sections were incubated without a primary antibody [7,143].

Samples were viewed under a microscope equipped with epifluorescence using appropriate filters (Leica DMR-XA; Leica Microsystems, Barcelona, Spain). Using a digital camera coupled to the microscope (FMVU-13S2CCS, Point Gray Research, Richmond, BC, Canada), images (×200) were captured from dorsal and ventral horns of GFAP- and Iba-1-immunostained histological sections. A minimum of five histological sections from each animal were evaluated. Images were analyzed using NIH Image software (ImageJ; version 1.37; National Institutes of Health, Bethesda, MD, USA), and GFAP immunostained histological sections were used to determine the area of immunoreactivity to GFAP, as an index of the degree of astrogliosis [143], whereas from Iba-1 immunostained histological sections, the percentage of reactive and nonreactive microglial cells was also determined, according their morphology. Nonreactive microglia cells show a ramified morphology, whereas reactive microglia show shorter thicker processes and an amoeboid shape [148,149]. The percentage of reactive microglia cells has been used previously as an index of the degree of microgliosis [7].

#### 3.4.3. Statistical Analysis

All functional and histological analyses were performed in a blinded manner using a numeric code for each mouse. The normal distribution of the data was analyzed by the Kolmogorov–Smirnov test before further applying parametric or nonparametric statistical analyses. When data followed a normal distribution, repeated measures MANOVA (Wilks’ criterion) and analysis of variance (ANOVA) followed by a Duncan’s test when applicable were used for data analysis. On the other hand, when data did not follow a normal distribution, they were analyzed by means of the Friedman statistical test for nonparametric repeated measures and a Kruskal–Wallis test, followed by a Bonferroni post hoc test. The percentage of antihyperalgesic effect exerted by a treatment was calculated as follows: % effect = [(PWD − PWV)/(PWN − PWV)] × 100, where PWD and PWV are the paw withdrawal latency (s) in drug-treated and pretreated animals, respectively, and PWN is the paw withdrawal in naïve animals. Pre- and post-pharmacological analyses were performed by the Wilcoxon test. In all analyses, the significance level α was set at 0.05, and the statistical program used was IBM SPSS Statistics 25.0 for Windows (IBM Corp. Released 2017; Armonk, NY, United States).

## 4. Conclusions

Repeated administration of BD1063 provided longer pain alleviation than pregabalin in the reserpine-induced myalgia model, whereas in the acidified saline intramuscular injection model, both treatments provided long-term nociplastic pain relief. In both fibromyalgia-like models, reflexive pain responses were accompanied by depression-like behaviors, which were alleviated by both drug treatments. Significant reductions in spinal cord gliosis were observed with BD1063 treatment in both experimental models of fibromyalgia. In summary, this is the first study in which the effect of a σ1R antagonist (BD1063) has been assessed in two fibromyalgia-like experimental models, observing significant thermal hyperalgesia alleviation and depression-like behavior, as well as a reduction in spinal cord gliosis. On the basis of these results, repeated BD1063 treatment is suggested to be a potential pharmacological strategy to alleviate both reflexive and nonreflexive pain responses present in fibromyalgia syndrome.

## Data Availability

All data generated or analyzed during this study are included in this published article.
